# Prevalence and outcomes of patients taking oral corticosteroids for over 1 month undergoing major surgery in England 2010–2020

**DOI:** 10.1111/anae.16532

**Published:** 2025-01-07

**Authors:** Jessica Harris, Georgina Russell, Barnaby Reeves, Ben Gibbison

**Affiliations:** ^1^ Bristol Trials Centre, Bristol Medical School University of Bristol Bristol UK; ^2^ Department of Endocrinology North Bristol NHS Trust Bristol UK; ^3^ Bristol Heart Institute, Bristol Medical School, University of Bristol Bristol UK

**Keywords:** adrenal gland, corticosteroids, glucocorticoids, routinely collected data, surgery

## Abstract

**Introduction:**

Approximately 1% of the UK population is prescribed oral corticosteroids at any one time. It is not known how many of these patients present for major surgery. We aimed to establish the prevalence, characteristics and outcomes of patients taking oral corticosteroids.

**Methods:**

We identified patients aged > 18 y undergoing major surgery between 1 April 2010 and 31 March 2020 from Hospital Episode Statistics with linked Clinical Practice Research Datalink data and the Office for National Statistics Mortality register in England. Prescribing data were used to define three sets of patients: ‘low‐dose’ – taking ≤ 7.5 mg oral prednisolone equivalents per day for at least 28/91 days before surgery; ‘high‐dose’ – taking > 7.5 mg oral prednisolone equivalents per day for at least 28/ 91 days before surgery; and a ‘no‐steroids’ group. We used ≤ 7.5 mg of prednisolone equivalents per day as our threshold, as this would likely exclude almost all patients who were taking corticosteroids as replacement for absolute adrenal/pituitary deficiency.

**Results:**

We identified 1,999,326 adult patients for inclusion in the dataset: 1,929,291 (96.5%) in the no‐steroids; 63,353 (3.2%) in the low‐dose group; and 6682 (0.3%) in the high‐dose group. Median (IQR [range]) duration of hospital stay increased with increasing dose of corticosteroid (no‐steroid 3 (0–14 [0–14,739]); low‐dose 5 (1–26 [1–8079]); and high‐dose 7 (2–28 [0–6956]) days). Mortality after the index surgery was 1.5%, 3.8% and 8.9% at 30 days and 5.5%, 11.6% and 39.9% at 1 year for no‐steroids, low‐dose and high‐dose groups, respectively.

**Conclusion:**

Around 1 in 29 patients undergoing major surgery are taking oral corticosteroids for > 28 days in the 3 months before major surgery. Their outcomes are poor and warrant highlighting within care pathways to aid risk prediction and mitigation.

## Introduction

Patients prescribed corticosteroids comprise those who are either: deficient in corticosteroids who require replacement (i.e. primary adrenal insufficiency and secondary adrenal insufficiency due to pituitary dysfunction); or those taking therapeutic corticosteroids for a range of inflammatory conditions (e.g. asthma and autoimmune diseases). The largest group is those taking therapeutic corticosteroids. The Association of Anaesthetists and the Society for Endocrinology, with support from the Royal College of Anaesthetists and the Royal College of Physicians, have been produced consensus guidelines for the management patients who take corticosteroids in the peri‐operative period [1].

There is no doubt that those patients with absolute deficiency due to adrenal or pituitary failure require supplementation in the peri‐operative period. The evidence for supplementation in those who are taking corticosteroids for anti‐inflammatory reasons is less clear [2, 3, 4]. The guidelines highlighted the lack of robust evidence on which to base management [1]. Our group has produced evidence from an audit [5] and a survey [6] showing that there is wide variation in the care given to patients who are taking oral corticosteroids, even after publication of the guidelines.

Patients who take corticosteroids are likely to present for surgery more frequently than the general population due to the indications (e.g. bowel resection for inflammatory bowel disease, joint replacement for arthritis) and the consequences (e.g. bone fracture due to demineralisation, accelerated coronary artery disease) of corticosteroid use. However, there is an absence of evidence to characterise this population. There are around 7.5 million prescriptions a year in the UK for oral corticosteroids at a cost of over £36 million (€43 million $45 million) [7]. While the number and cost of prescriptions is known, it is not known to how many patients these prescriptions are given and the number of these patients who present for major surgery. To gain information to design future studies, we carried out an analysis of an English 10‐year cohort with linked GP and hospital care data to describe the number of patients, the number of these who present for major surgery taking oral corticosteroids and their characteristics and outcomes up to a year after surgery.

## Methods

This was a retrospective cohort study of patients using Clinical Practice Research Datalink (CPRD), Hospital Episode Statistics and Office for National Statistics linked data. We included both CPRD Gold and Aurum datasets and extracted data from the admitted patient care subset of Hospital Episode Statistics. We used the Office for National Statistics mortality register data. Clinical Practice Research Datalink Gold covers around 21 million patients, of whom around 3 million are currently alive and contributing data (5% of the population of England) [8]. Clinical Practice Research Datalink Aurum covers 19 million patients, of whom about 7 million are alive and currently contributing (13% of the population of England) [9].

We requested linked data for adult (aged ≥ 18 y) patients undergoing major surgery which was performed or funded by the NHS between 1 April 2010 and 31 March 2020. We requested data from 1 April 2009 to 31 March 2021 to provide a 10‐year cohort of patients, with data for the 12 months before and after surgery. Changes in access to and provision of healthcare changed with the onset of the COVID‐19 pandemic in March 2020 and therefore we stopped entry to the cohort on 31 March 2020. We used a previously established and validated set of OPCS‐4 codes [10] to identify major and extra‐major surgical procedures. When applied to the whole English population, these procedure codes identified around 1.2 million procedures per year.

Inclusion criteria were: age 18 y or over at the time of the index surgery; acceptable user status registered in CPRD, registered at an up‐to‐standard practice and eligible for the CPRD linkage scheme; and at least 12 months of up‐to‐standard pre‐operative follow‐up. Those patients with an index surgical procedure before 1 April 2010 or after 31 March 2020 and those not registered at the GP practice for at least one year before their index surgical procedure were not studied.

Patients entered the cohort 12 months before an eligible OPCS‐4 code was first recorded and were followed up until 31 March 2020 or earlier (if they died or were transferred out of their primary care practice at the time of surgery). We used prescribing data from CPRD to define three sets of patients: ‘low‐dose’ – those taking ≤ 7.5 mg oral prednisolone equivalents per day for at least 28 days in the 91 days (3 months) before surgery; and ‘high‐dose’ – those taking > 7.5 mg oral prednisolone equivalents per day for at least 28 days in the 91 days before surgery. The final group was a set of patients who did not fulfil either of these criteria (‘no steroids’). We used 7.5 mg prednisolone equivalents per day as our threshold as this would likely exclude almost all patients who were taking corticosteroids as replacement for absolute adrenal/pituitary deficiency (25 mg hydrocortisone – towards the top end of daily replacement dose – which is approximately equal to 6.5 mg oral prednisolone equivalents). We performed a sensitivity analysis to ensure that patients taking oral corticosteroids for < 28 days did not affect the results by not including these patients from the no‐steroids group.

We were provided with patient characteristics, the Index of Multiple Deprivation quintile and surgical urgency. We calculated the oral prednisolone equivalents per day using National Institute of Health and Care Excellence oral corticosteroid equivalence tables [11], as well as the age‐adjusted Charlson comorbidity index. The Charlson comorbidity index was calculated as the maximum score derived from CPRD data [12] and from Hospital Episode Statistics data [13]. Body mass index values were calculated from the most recent height and weight measurements, and values outside the plausible range of 10–70 were omitted. All diagnoses recorded in episodes in the 12 months before the index admission date and the index admission episode contributed to derivation of the Charlson comorbidity index. This assumed the patient did not have any of the risk factors. The surgical specialty for the index surgery was categorised using the higher level OPCS‐4 codes [14].

We extracted the duration of hospital stay (defined as the continuous inpatient stay including and after the index surgery); mortality at 30 days and 1 year after the index surgery; and the days alive and out of hospital at 30 days. We also calculated the number of admissions to hospital and the number of primary‐care consultations in the year following the index surgery. We described 11 pre‐specified postoperative outcomes in the 30 days following the index surgery such as acute coronary syndrome; pneumonia; and acute kidney injury.

## Results

We identified 1,999,326 patients from 1 April 2010 to 31 March 2020 who were eligible for inclusion in the dataset (i.e. had eligible data and had an episode of major surgery). A total of 1,929,291 (96.5%) patients were in the no‐steroids group, 63,353 (3.2%) in the low‐dose group and 6682 (0.3%) in the high‐dose group. The no‐steroids group included 14,220 patients who were prescribed oral corticosteroids, but not for > 28 days (see online Supporting Information Tables S1–S3). Median (IQR [range]) daily dose of corticosteroids (in oral prednisolone equivalents) was no‐steroids 0 mg; low‐dose 5 (5.0–5.0 [0.2–6.7]) mg; high‐dose 13.3 (10.0–13.3 [7.5–106.7]) mg.

Patient characteristics for all three groups are detailed in Table 1. Patients who took corticosteroids for > 28 days in the 91 days before surgery were, on average, older (no‐steroids 59 y; low‐dose 68 y; and high‐dose 65 y) and more likely to be of White ethnicity (no‐steroids 88.9%; low‐dose 92.6%; and high‐dose 92.5%) than those in the no‐steroids group. Most patients were having elective surgery, although the low‐dose and high‐dose groups had an incrementally lower proportion of elective surgery than those in the no‐steroids group (no‐steroids 74.3%; low‐dose 70.5%; high‐dose 67.1%). Patients had a small incremental increase in Charlson comorbidity index with increasing does of corticosteroids (median (IQR [range]) no‐steroids 2 (0–4 [0–23]), low‐dose 4 (2–6 [0–21]); and high‐dose 5 (3–9 [0–18])).

**Table 1:** Pre‐operative characteristics of patients stratified by use of oral corticosteroids. Values are median (IQR [range]) or number (proportion).

	No‐steroids	Low‐dose steroids > 28 days	High‐dose steroids > 28 days	Total
	n = 1,929,291	n = 63,353	n = 6682	n = 1,999,326
Prednisolone daily dose;mg^1^	0	5.0 (5.0–5.0 [0.2–6.7])	13.3 (10.0–13.3 [7.5–106.7])	0.0 (0.0–0.0 [0.2–106.7])
Age; y	59 (44–72 [18–112])	68 (57–77 [18–105])	65 (54–73 [18–100])	60 (45–72 [18–112])
Sex; female^2^	1,127,109 (58.4%)	39,203 (61.9%)	3593 (53.8%)	1,169,905 (58.5%)
Ethnicity^3^				
Bangladeshi	5867 (0.3%)	124 (0.2%)	11 (0.2%)	6002 (0.3%)
Black African	18,638 (1.0%)	296 (0.5%)	42 (0.6%)	18,976 (0.9%)
Black Caribbean	19,306 (1.0%)	484 (0.8%)	36 (0.5%)	19,826 (1%)
Black Other	9557 (0.5%)	161 (0.3%)	19 (0.3%)	9737 (0.5%)
Chinese	4814 (0.2%)	77 (0.1%)	14 (0.2%)	4905 (0.2%)
Indian	29,403 (1.5%)	868 (1.4%)	69 (1%)	30,340 (1.5%)
Mixed	12,546 (0.7%)	255 (0.4%)	26 (0.4%)	12,827 (0.6%)
Asian Other	18,031 (0.9%)	388 (0.6%)	34 (0.5%)	18,453 (0.9%)
Other	26,105 (1.4%)	465 (0.7%)	54 (0.8%)	26,624 (1.3%)
Pakistani	17,654 (0.9%)	532 (0.8%)	44 (0.7%)	18,230 (0.9%)
Unknown	37,310 (1.9%)	504 (0.8%)	100 (1.5%)	37,914 (1.9%)
White	1,714,311 (88.9%)	58,639 (92.6%)	6182 (92.5%)	1,779,132 (89.0%)
BMI^4^; kg.m^‐2^	27.0 (23.7–31.0 [10.0–70.0])	27.2 (23.7–31.4 [11.5–70.0])	26.5 (23.2–30.4 [23.2–30.4])	27.0 (23.7–31.0 [10–70])
Index of Multiple Deprivation^5^				
1	404,374 (21.0%)	12,825 (20.2%)	1440 (21.6%)	418,639 (20.9%)
2	410,179 (21.3%)	12,869 (20.3%)	1564 (23.4%)	424,612 (21.2%)
3	384,449 (19.9%)	12,572 (19.8%)	1361 (20.4%)	398,382 (19.9%)
4	375,957 (19.5%)	12,317 (19.4%)	1208 (18.1%)	389,482 (19.5%)
5	352,823 (18.3%)	12,722 (20.1%)	1104 (16.5%)	366,649 (18.3%)
Surgery^6^				
Elective	1,433,998 (74.3%)	44,687 (70.5%)	4486 (67.1%)	1,483,171 (74.2%)
Emergency	467,752 (24.2%)	17,619 (27.8%)	2045 (30.6%)	48,7416 (24.4%)
Maternity	5136 (0.3%)	37 (0.1%)	0	5173 (0.3%)
Other	22,232 (1.2%)	1002 (1.6%)	150 (2.2%)	23,384 (1.2%)
Charlson comorbidity index	2 (0–4 [0–23])	4 (2–6 [0–21])	5 (3–9 [0–18])	2 (1–4 [0–23])

Data missing for ^1^1 patient; ^2^10 patients; ^3^16,360 patients; ^4^249,682 patients; ^5^1562 patients; ^6^182 patients.

The most common surgical specialties for the index surgery in the no‐steroids group were ‘other bones and joints’ (35.8%), ‘bones and joints of the skull and spine’ (8.1%) and ‘lower digestive system’ (7.4%). The most common surgical specialties for the index surgery in the low‐dose group were ‘other bones and joints’ (31.6%), ‘arteries and veins’ (11.7%) and ‘respiratory tract’ (9.1%). In the high‐dose group, the most common surgical specialties were ‘nervous system’ (33.9%), ‘other bones and joints’ (15.2%) and ‘arteries and veins’ (10.8%). Full details of the surgical specialties by group are in Table 2.

**Table 2:** Surgical specialties for index surgery in patients stratified by use of oral corticosteroids. Values are number (proportion).

OPCS‐4 chapter	No‐steroids	Low‐dose steroids > 28 days	High‐dose steroids > 28 days	Total
	(n = 1,929,291)	(n = 63,353)	(n = 6682)	(n = 1,999,326)
Nervous system	73,159 (3.8%)	2354 (3.7%)	2263 (33.9%)	77,776 (3.9%)
Endocrine system and breast	121,647 (6.3%)	2867 (4.5%)	140 (2.1%)	124,654 (6.2%)
Eye	1581 (0.1%)	64 (0.1%)	< 5 (< 0.1%)	
Ear	6280 (0.3%)	132 (0.2%)	5 (0.1%)	6417 (0.3%)
Respiratory tract	59,291 (3.1%)	5749 (9.1%)	534 (8.0%)	65,574 (3.3%)
Mouth	14,805 (0.8%)	402 (0.6%)	21 (0.3%)	15,228 (0.8%)
Upper digestive system	63,655 (3.3%)	2766 (4.4%)	346 (5.2%)	66,767 (3.3%)
Lower digestive system	143,346 (7.4%)	4435 (7.0%)	481 (7.2%)	148,262 (7.4%)
Other abdominal organs, principally digestive	136,023 (7.1%)	2818 (4.4%)	131 (2.0%)	138,972 (7.0%)
Heart	68,068 (3.5%)	2436 (3.8%)	85 (1.3%)	70,589 (3.5%)
Arteries and veins	74,436 (3.9%)	7425 (11.7%)	720 (10.8%)	82,581 (4.1%)
Urinary	39,050 (2.0%)	1078 (1.7%)	66 (1.0%)	40,194 (2.0%)
Male genital organs	7363 (0.4%)	123 (0.2%)	9 (0.1%)	7495 (0.4%)
Lower female genital tract	18,282 (0.9%)	311 (0.5%)	13 (0.2%)	18,606 (0.9%)
Upper female genital tract	141,361 (7.3%)	2025 (3.2%)	82 (1.2%)	143,468 (7.2%)
Skin	4925 (0.3%)	82 (0.1%)	< 5 (< 0.1%)	
Soft tissue	95,976 (5.0%)	3253 (5.1%)	329 (4.9%)	99,558 (5.0%)
Bones and joints of skull and spine	156,011 (8.1%)	4554 (7.2%)	406 (6.1%)	160,971 (8.1%)
Other bones and joints	690,374 (35.8%)	20,019 (31.6%)	1016 (15.2%)	711,409 (35.6%)
Miscellaneous operations	13,658 (0.7%)	460 (0.7%)	27 (0.4%)	14,145 (0.7%)

Counts < 5 are suppressed to maintain patient confidentiality.

OPCS‐4, Office of Population Censuses and Surveys classification system for interventions and surgical procedures.

The median (IQR [range]) duration of hospital stay rose with increasing dose of steroids (no‐steroids 3 (0–14 [0–14,739]) days; low‐dose 5 (1–26 [1–8079]) days; and high‐dose 7 (2–28 [0–6956]) days). Mortality at 30 days and 1 year after the index surgery rose with increasing dose of steroids (1.5%, 3.8% and 8.9% at 30 days and 5.5%, 11.6% and 39.9% at 1 year for no‐steroids, low‐dose and high‐dose groups, respectively). The mortality rate by specialty (online Supporting Information Table S4) was similar to the proportions of surgery by specialty (Table 2). There was a higher incidence of pneumonia in the groups receiving corticosteroids (1.2%, 3.3% and 4.9% for no‐steroids, low‐dose and high‐dose groups, respectively). Rates of surgical site infection were similar in each group. Full details of all outcomes are in Table 3.

**Table 3:** Outcomes of patients undergoing major surgery stratified by use of oral corticosteroids. Values are median (IQR [range]) and number (proportion).

	No‐steroids	Low‐dose steroids > 28 days	High‐dose steroids > 28 days	Total
	n = 1,929,291	n = 63,353	n = 6682	n = 1,999,326
Duration of hospital stay^1^; d	3 (0–14 [0–14,739])	5 (1–26 [1–8079])	7 (2–28 [0–6956])	3 (0–14 [0–14,739])
Mortality at 30 days	28,921 (1.5%)	2422 (3.8%)	595 (8.9%)	31,938 (1.6%)
30 days alive and out of hospital	1,671,312 (86.6%)	51,500 (81.3%)	5090 (76.2%)	172,7902 (86.4%)
Mortality at 1 year	106,822 (5.5%)	7628 (11.6%)	2669 (39.9%)	117,119 (5.9%)
Readmission within 30 days	155,504 (8.1%)	7330 (11.6%)	1352 (20.2%)	164,186 (8.2%)
Number of admissions in year after surgery	0 (0–1 [0–275])	1 (0–2 [0–168])	1 (0–2 [0–164])	0 (0–1 [0–275])
Number of GP consultations in year after surgery	8 (3–14 [0–365])	13 (7–22 [0–311])	12 (5–21 [0–158])	8 (3–14 [0–365])
**30‐day outcomes**				
Myocardial infarction	5275 (0.3%)	321 (0.5%)	16 (0.2%)	5612 (0.3%)
Acute coronary syndrome	6962 (0.4%)	432 (0.7%)	24 (0.4%)	7418 (0.4%)
Cardiac arrest within 30 days	2123 (0.1%)	153 (0.2%)	14 (0.2%)	2290 (0.1%)
Pulmonary embolism	4503 (0.2%)	263 (0.4%)	124 (1.9%)	4890 (0.2%)
Atrial fibrillation	35,928 (1.9%)	2252 (3.6%)	190 (2.8%)	38,370 (1.9%)
Acute kidney injury	19,269 (1.0%)	1192 (1.9%)	110 (1.6%)	20,571 (1.0%)
Stroke	8390 (0.4%)	312 (0.5%)	56 (0.8%)	8758 (0.4%)
Surgical site infection	18,514 (1.0%)	789 (1.2%)	99 (1.5%)	19,402 (1.0%)
Pneumonia	23,993 (1.2%)	2075 (3.3%)	327 (4.9%)	26,395 (1.3%)
Delirium	6189 (0.3%)	277 (0.4%)	35 (0.5%)	6501 (0.3%)
Paralytic ileus	500 (< 0.1%)	27 (< 0.1%)	< 5 (< 0.1%)	

Counts < 5 are suppressed to maintain patient confidentiality.

^1^ Data missing for 3 patients.

Patients had a median (IQR [range]) of one admission in the year following surgery in the low‐dose (1 (0–2) [0–168]) and high‐dose groups (1 (0–2 [0–164]), compared with a median of none in the no‐steroids group (0 (0–1) [0–275]). Patients in the low‐dose and high‐dose groups had more contact with primary care in the year following surgery (13 (7–22) [0–311]) consultations and 12 (5–21 [0–158]) consultations, respectively) than those in the no‐steroids group (8 (3–14 [0–365]) consultations).

Performing the analysis after not including patients who were taking oral corticosteroids, but not for > 28 days, made no meaningful difference to the results for the no‐steroids group (see online Supporting Information Table S3). Performing the analysis on 1‐year outcomes after omitting patients with an index surgery between 1 April 2019 and 31 March 2020 (whose follow‐up may have been affected by COVID‐19) also made no meaningful difference to the outcomes (see online Supporting Information Table S5).

## Discussion

This retrospective cohort study has shown that around 3.5% (1 in 29) of the population undergoing major surgery in England was taking oral corticosteroids for > 28 days in the 3 months before surgery. Higher doses of corticosteroids are associated with increases in duration of hospital stay and mortality (both at 30 days and 1 year). In those taking the highest doses of oral corticosteroids, 40% (1 in 2.5 patients) will have died within 1 year. Even in those taking what are considered modest doses of oral corticosteroid (≤ 7.5 mg oral prednisolone equivalent per day for 28 days), 12% (1 in 8 patients) will have died within a year of the surgery. Patients taking higher doses of oral corticosteroids undergo surgery in different specialties from those taking lower‐dose and no corticosteroids.

The study is descriptive only and implies no causal effect of oral corticosteroids on peri‐operative outcomes. However, there appears to be a clear association between increasing doses of steroids and worsening peri‐operative and 1‐year outcomes. It is likely that, at least in part, higher doses of oral corticosteroids are indicative of more severe disease, which may lead to worse outcomes (although we cannot measure or eliminate the effect of corticosteroids on outcomes in this study).

We are unable to unpick how much of the effect is suboptimal peri‐operative management. However, given that the majority of mortality occurs beyond 30 days, this is unlikely to be significant factor. One‐third of patients on the highest doses of corticosteroids were having neurosurgery, but the proportion of patients who died analysed by surgical specialty are broadly in line with the proportion of surgery by surgical specialty. This implies that patients taking corticosteroids are at high‐risk of morbidity and mortality regardless of the type of surgery.

It does not appear that oral corticosteroids are a risk factor for infection per se, in that the rates of surgical site infection were equal between the groups. However, there was an incremental rise in the frequency of pneumonia with increasing corticosteroid dose. This again may represent significant comorbidity rather than immunosuppression (as reflected in the higher Charlson comorbidity index in those patients taking corticosteroids).

The strength of this study is that it used unselected patient data and therefore gives a ‘real‐world’ estimate of the proportion of patients taking oral corticosteroids when undergoing major surgery in England. Hospital Episode Statistics data contain all episodes of secondary care in England and so are likely to be representative. Clinical Practice Research Datalink data are broadly representative of the UK population [9, 15], but in recent years have shown a slightly higher proportion of GP practices in deprived areas [16] and a higher than expected proportion of minority ethnic groups [17]. Linking two large datasets (one primary care with detailed community prescribing, and one secondary care with detailed diagnostics and outcomes) allows high precision in these estimates across a population. The small number of large (and spurious) values seen in the ranges (but not IQR) for durations of hospital stay and follow‐up appointments are due to this being ‘real‐world’ data and being inadequately validated and cleaned at source. Using medians and IQRs gives a good representation of these parameters at high precision for the population. This dataset will miss hospital‐based outpatient prescribing of corticosteroids which may cause an underestimate and it may include patients prescribed rescue corticosteroids for ‘flare‐ups’ (e.g. exacerbation of chronic obstructive pulmonary disease) which were never taken, leading to some degree of overestimation. We also do not know what the indication for the oral corticosteroids was. Overall, the findings are likely to be broadly accurate.

We included major surgery in this dataset and so we do not know what the proportion is of patients presenting for all severities of surgery. Our research question aimed to describe the characteristics of patients taking corticosteroids for inflammatory reasons who may need supplementing during the peri‐operative period, the procedures they were having and their outcomes. However, since the probability of adrenal crisis after minor surgery in those at risk of tertiary hypoadrenalism is low, their inclusion would not have been useful in answering the question. This cohort also does not include those patients having caesarean sections or in labour. Maternity admissions in this cohort were patients who were, or recently were, pregnant and had major surgery. Another weakness is that patients will continue to contribute to the denominator (the population), without contributing to the numerator (number of primary care interactions/readmissions) because we could not censor patients from follow‐up outcomes (i.e. hospital admission, primary care interactions) after they had died. This is a potential explanation for the fewer primary care interactions in the high‐dose group.

There will have been some change in care for following up patients having surgery during 2019–2020 because of the effects of COVID‐19 on healthcare provision after early 2020. The effects of these on 30‐day outcomes are likely to be small and our analysis showed no difference in the 1‐year outcomes when patients having their index surgery during this period were omitted.

Previous work has shown that around 1% of the UK population is taking oral corticosteroids [18] and our scoping work for this project within the CPRD dataset confirmed that this estimate is still correct [19]. What has not been reported previously is the number of these patients who present for major surgery and may require supplementation. This cohort study answers that question. There is still much debate about which patients taking oral corticosteroids need supplementing and if so, how much and for how long. It is clear that those with primary adrenal and primary pituitary disease (not included in this cohort) all need extra corticosteroid in the peri‐operative period. The evidence for patients taking exogenous steroids for anti‐inflammatory and other causes and who are at risk of tertiary hypoadrenalism is less clear. Many studies suggest that these patients do not need supplementation on top of their routine daily dose. The dose threshold for supplementation also varies between the UK (prednisolone 5 mg equivalent daily) and USA (prednisolone 20 mg equivalent daily). A very small number of patients in our dataset would reach the threshold for peri‐operative supplementation in the USA.

The high‐, short‐ and mid‐term mortality from this cohort study suggests that patients taking oral corticosteroids for > 28 days and having major surgery warrants further study. Since 1 in 29 patients undergoing all major surgery would be eligible for recruitment, it is likely that large scale, randomised trials of supplementation regimens would be feasible. This would only answer questions of peri‐operative supplementation, but not those of patient selection, shared decision‐making, optimisation and rehabilitation. Work should also continue using routinely‐collected data (to be as inclusive and ensure sufficient generalisability) to establish whether it is the corticosteroids, the background condition, variations in peri‐operative supplementation [5] or a combination of these that impacts on clinical outcomes. This would allow more accurate risk prediction and mitigation for those taking oral corticosteroids and contemplating major surgery.

In our previous work, we have shown that there is significant variation in UK practice in terms of who is supplemented in the peri‐operative period, what they are given and for how long. Most practitioners do not follow the published national guidelines [5, 6] despite saying (or at least thinking) that they are doing so. Given this situation, guidelines should focus on identifying the patients who require supplementation. A reasonable approach for the future should be implementation research. Implementation research is “*the scientific inquiry into questions concerning implementation – the act of carrying an intention into effect, which in health research can be policies, programmes, or individual practices*” [20]. In this case, that means finding the best solutions that allow clinicians to identify at‐risk individuals and develop and embed a consensus‐driven treatment strategy agreed and used by all stakeholders (e.g. researchers, patients, practitioners and managers). This would reduce variation in care and improve what appear to be very poor outcomes.

The implications of this research for clinicians are that patients within the surgical care pathway who are taking oral corticosteroids for > 1 month before their surgery should be highlighted as high‐risk. Shared decision‐making should be implemented so that patients and practitioners embark on the surgery aware of each other's wants and expectations. Given the high prevalence of patients taking oral corticosteroids, funding bodies should consider supporting studies which seek to understand why the clinical outcomes in this group of patients are so poor and then move to large scale pragmatic trials of interventions that seek to improve those outcomes.

In conclusion, this cohort study has shown that around 1 in 29 patients (3.5%) of patients undergoing major surgery in England are taking oral corticosteroids for > 1 month. Patients taking oral corticosteroids have a significantly increased risk of mortality and morbidity in the short and mid‐term after major surgery.

## Supporting information


**Table S1.** Pre‐operative characteristics of patients, with those on oral corticosteroids for < 28 days removed from the analysis.
**Table S2.** Surgical specialties for index surgery of patients, with those on oral corticosteroids for < 28 days removed from the analysis.
**Table S3.** Outcomes of patients, with those on oral corticosteroids for < 28 days removed from the analysis.
**Table S4.** Mortality by surgical specialties for index surgery.
**Table S5.** One‐year outcomes not including those with a surgery date between 1 April 2019 and 31 March 2020.
