# Supplementary material for: Prevalence and outcomes of patients taking oral corticosteroids for over 1 month undergoing major surgery in England 2010–2020
Source: Anaesthesia. 2025 Jan 7;80(4):404–11. doi: 10.1111/anae.16532 (PMC11885188; doi:10.1111/anae.16532)
Supplement: Supplementary file 1 — Table S1. Pre‐operative characteristics of patients, with those on oral corticosteroids for < 28 days removed from the analysis. Table S2. Surgical specialties for index surgery of patients, with those on oral corticosteroids for < 28 days removed from the analysis. Table S3. Outcomes of patients, with those on oral corticosteroids for < 28 days removed from the analysis. Table S4. Mortality by surgical specialties for index surgery. Table S5. One‐year outcomes not including those with a surgery date between 1 April 2019 and 31 March 2020. [file ANAE-80-404-s001.docx]

# Table S1: Pre-operative characteristics of patients with those patients on oral corticosteroids for <28 days removed from the analysis. Values are median (IQR[range]) and number (proportion).

|  |  | **No steroids**  **(n=1,915,071)** | **Low dose**  **>28 days**  **(n=63,353)** | **High dose > 28 days**  **(n=6,682)** | **Total**  **(n=1,985,106)** |
| --- | --- | --- | --- | --- | --- |
| Prednisolone.day ^-1^ |  | 0.0 (0.0, 0.0 [0.0, 0.0]) | 5.0 (5.0, 5.0 [0.2, 6.7]) | 13.3 (10.0, 13.3 [7.5, 106.7]) | 0.0 (0.0, 0.0 [0.2, 106.7]) |
| Age; y |  | 59 (44, 72 [18, 112]) | 68 (57, 77[18, 105]) | 66 (54, 73 [18, 100]) | 60 (45, 72 [18, 112]) |
| Sex ^2^ | Male | 796,931 (42%) | 24,150 (38%) | 3,089 (46%) | 824,170 (42%) |
|  | Female | 1,118,130 (58%) | 39,203 (62%) | 3,593 (54%) | 1,160,926 (58%) |
| Ethnicity ^3^ | Bangladeshi | 5,832 (0.3%) | 124 (0.2%) | 11 (0.2%) | 5,967 (0.3%) |
|  | Black African | 18,550 (1%) | 296 (0.5%) | 42 (1%) | 18,888 (1%) |
|  | Black Caribbean | 19,168 (1%) | 484 (1%) | 36 (1%) | 19,688 (1%) |
|  | Black Other | 9,512 (1%) | 161 (0.3%) | 19 (0.3%) | 9,692 (0.5%) |
|  | Chinese | 4,799 (0.3%) | 77 (0.1%) | 14 (0.2%) | 4,890 (0.3%) |
|  | Indian | 29,145 (2%) | 868 (1%) | 69 (1%) | 30,082 (2%) |
|  | Mixed | 12,487 (1%) | 255 (0.4%) | 26 (0.4%) | 12,768 (1%) |
|  | Asian Other | 1,7924 (1%) | 388 (1%) | 34 (1%) | 18,346 (1%) |
|  | Other | 25,986 (1%) | 465 (1%) | 54 (1%) | 26,505 (1%) |
|  | Pakistani | 17,482 (1%) | 532 (1%) | 44 (1%) | 18,058 (1%) |
|  | Unknown | 37,191 (2%) | 504 (1%) | 100 (2%) | 37,795 (2%) |
|  | White | 1,701,346 (90%) | 58,639 (93%) | 6,182 (93%) | 1,766,167 (90%) |
| BMI ^4^ |  | 27.0 (23.7, 31.0 [10.0, 70.0) | 27.2 (23.7, 31.4 [11.5, 70]) | 26.5 (23.2, 30.4 [23.2, 30.4]) | 27.0 (23.7, 31.0 [10, 70]) |
| IMD ^5^ [1=least deprived | 1 | 401,552 (21%) | 12,825 (20%) | 1,440 (22%) | 415,817 (21%) |
|  | 2 | 407,084 (21%) | 12,869 (20%) | 1,564 (23%) | 421,517 (21%) |
|  | 3 | 381,499 (20%) | 12,572 (20%) | 1,361 (20%) | 395,432 (20%) |
|  | 4 | 373,202 (20%) | 12,317 (19%) | 1,208 (18%) | 386,727 (19%) |
|  | 5 | 350,241 (18%) | 12,722 (20%) | 1,104 (17%) | 364,067 (18%) |
| Surgical urgency ^6^ | Elective | 1,424,440 (74%) | 44,687 (71%) | 4,486 (67%) | 1,473,613 (74%) |
|  | Emergency | 463,316 (24%) | 17,619 (28%) | 2,045 (31%) | 482,980 (24%) |
|  | Maternity | 5,128 (0.3%) | 37 (0.1%) | 0 (0%) | 5,165 (0.3%) |
|  | Other | 22,014 (1%) | 1,002 (2%) | 150 (2%) | 23,166 (1%) |
| Charlson Comorbidity Index |  | 2 (0, 4 [0, 23]) | 4 (2, 6 [0, 21]) | 5 (3, 9 [0, 18]) | 2 (1, 4 [0, 23]) |

Missing data: ^1^ missing for 1 patient; ^2^ missing for 10 patients; ^3^ missing for 16,260 patients; ^4^ missing for 248,311 patients; ^5^ missing for 1,546 patients; ^6^ missing for 182 patients*.* BMI, body mass index; IMD, index of multiple deprivation.

# Table S2: Surgical Specialties for Index Operation of patients with those patients on oral corticosteroids for <28 days removed from the analysis. Values are number (proportion)

| **OPCS chapter** | **No steroids**  **(n=1,915,071)** | **Low dose**  **>28 days**  **(n=63,353)** | **High dose > 28 days**  **(n=6,682)** | **Total**  **(n=1,985,106)** |
| --- | --- | --- | --- | --- |
| Nervous system | 72335 (4%) | 2354 (4%) | 2263 (34%) | 76952 (4%) |
| Endocrine system and breast | 121054 (6%) | 2867 (5%) | 140 (2%) | 124061 (6%) |
| Eye | 1567 (0.1%) | 64 (0.1%) | <5 |  |
| Ear | 6258 (0.3%) | 132 (0.2%) | 5 (0.1%) | 6395 (0.3%) |
| Respiratory tract | 58366 (3%) | 5749 (9%) | 534 (8%) | 64649 (3%) |
| Mouth | 14636 (1%) | 402 (1%) | 21 (0.3%) | 15059 (1%) |
| Upper digestive system | 63104 (3%) | 2766 (4%) | 346 (5%) | 66216 (3%) |
| Lower digestive system | 142527 (7%) | 4435 (7%) | 481 (7%) | 147443 (7%) |
| Other abdominal organs, principally digestive | 135455 (7%) | 2818 (4%) | 131 (2%) | 138404 (7%) |
| Heart | 67600 (4%) | 2436 (4%) | 85 (1%) | 70121 (4%) |
| Arteries and veins | 73233 (4%) | 7425 (12%) | 720 (11%) | 81378 (4%) |
| Urinary | 38744 (2%) | 1078 (2%) | 66 (1%) | 39888 (2%) |
| Male genital organs | 7331 (0.4%) | 123 (0.2%) | 9 (0.1%) | 7463 (0.4%) |
| Lower female genital tract | 18205 (1%) | 311 (0.5%) | 13 (0.2%) | 18529 (1%) |
| Upper female genital tract | 140915 (7%) | 2025 (3%) | 82 (1%) | 143022 (7%) |
| Skin | 4893 (0.3%) | 82 (0.1%) | <5 |  |
| Soft tissue | 95257 (5%) | 3253 (5%) | 329 (5%) | 98839 (5%) |
| Bones and joints of skull and spine | 154943 (8%) | 4554 (7%) | 406 (6%) | 159903 (8%) |
| Other bones and joints | 685134 (36%) | 20019 (32%) | 1016 (15%) | 706169 (36%) |
| Miscellaneous operations | 13514 (1%) | 488 (1%) | 27 (0.4%) | 14848 (1%) |

OPCS-4, Office of Population Censuses and Surveys classification system for interventions and surgical procedures

**Table S3:** Outcomes of patients with those patients on oral corticosteroids for <28 days removed from the analysis. Values are median (IQR[range]) and number (proportion).

|  |  | **No steroids**  **(n=1,915,071)** | **Low dose**  **>28 days**  **(n=63,353)** | **High dose > 28 days**  **(n=6,682)** | **Total**  **(n=1,985,106)** |
| --- | --- | --- | --- | --- | --- |
| Hospital length of stay (continuous inpatient spell) |  | 3 (0, 14 [0, 14739]) | 5 (1, 26 [0, 8079]) | 7 (2, 28 [0, 6956]) | 3 (0, 14 [0, 14739]) |
| Mortality at 30 days |  | 28,242 (1%) | 2,422 (4%) | 595 (9%) | 31,259 (2%) |
| 30 days alive and out of hospital |  | 1,660,195 (87%) | 51,500 (81%) | 5,090 (76%) | 1,716,785 (86%) |
| Mortality at 1 year |  | 104,321 (5%) | 7,628 (12%) | 2,669 (40%) | 114,618 (6%) |
| Readmission within 30 days |  | 153,783 (8%) | 7,330 (12%) | 1,352 (20%) | 162,465 (8%) |
| Number of admissions in year after surgery |  | 0 (0, 1 [0, 275]) | 1 (0, 2 [0, 168]) | 1 (0, 2 [0, 164]) | 0 (0, 1 [0, 275]) |
| Number of GP consultations in year after surgery |  | 8 (3, 14 [0, 365]) | 14 (7, 22 [0, 311]) | 12 (5, 21 [0, 158]) | 8 (3, 14 [0, 365]) |
| **30-day outcomes** | **OPCS codes** |  |  |  |  |
| Myocardial infarction | I21, I22 | 5,221 (0.3%) | 321 (0.5%) | 16 (0.2%) | 5,558 (0.3%) |
| Acute coronary syndrome | I20.0, I21, I22, I24.9 | 6,886 (0.4%) | 432 (1%) | 24 (0.4%) | 7,342 (0.4%) |
| Cardiac arrest within 30 days | I46 | 2,090 (0.1%) | 153 (0.2%) | 14 (0.2%) | 2,257 (0.1%) |
| Pulmonary embolism | I26 | 4,437 (0.2%) | 263 (0.4%) | 124 (2%) | 4,824 (0.2%) |
| Atrial fibrillation | I48 | 35,355 (2%) | 2,252 (4%) | 190 (3%) | 37,797 (2%) |
| Acute Kidney Injury | N17, N19 | 18,951 (1%) | 1,192 (2%) | 110 (2%) | 20,253 (1%) |
| Stroke | I60, I61, I62, I63, I64 | 8,313 (0.43%) | 312 (0.5%) | 56 (1%) | 8,681 (0.4%) |
| Surgical site infection | T81.4 | 18,343 (1%) | 789 (1%) | 99 (1%) | 19,231 (1%) |
| Pneumonia | J12, J18 | 23,517 (1%) | 2,075 (3%) | 327 (5%) | 25,919 (1%) |
| Delirium | F05 | 6,097 (0.3%) | 277 (0.4%) | 35 (1%) | 6,409 (0.3%) |
| Paralytic ileus | K56.0 | 492 (0.03%) | 27 (0.04%) | <5 |  |

Missing data: ^1^ missing for 3 patients.

**Table S4:** Mortality by Surgical Specialties for Index Operation. Counts <5 are suppressed to maintain patient confidentiality. Values are number (proportion).

| **OPCS-4 chapter** | **No-steroids**  **(n=106,822)** | **Low-dose steroids**  **>28 days**  **(n=7,268)** | **High-dose steroids**  **>28 days**  **(n=2,669)** | **Total**  **(n=117,119)** |
| --- | --- | --- | --- | --- |
| Nervous system | 6,209 (6%) | 252 (3%) | 988 (37%) | 7,449 (6%) |
| Endocrine system and breast | 1,718 (2%) | 124 (2%) | 22 (1%) | 1,864 (2%) |
| Eye | 89 (0.1%) | 9 (0.1%) | <5 |  |
| Ear | 56 (0.1%) | 5 (0.1%) | <5 |  |
| Respiratory tract | 10,182 (10%) | 1,296 (17%) | 340 (13%) | 11,818 (10%) |
| Mouth | 319 (0.3%) | 17 (0.2%) | 4 (0.1%) | 340 (0.3%) |
| Upper digestive system | 9,237 (9%) | 510 (7%) | 167 (6%) | 9,914 (8%) |
| Lower digestive system | 9,322 (9%) | 613 (8%) | 147 (6%) | 10,082 (9%) |
| Other abdominal organs, principally digestive | 2,702 (3%) | 144 (2%) | 21 (1%) | 2,867 (2%) |
| Heart | 4,362 (4%) | 293 (4%) | 18 (1%) | 4,673 (4%) |
| Arteries and veins | 8,271 (8%) | 925 (12%) | 203 (8%) | 9,399 (8%) |
| Urinary | 1,974 (2%) | 96 (1%) | 24 (1%) | 2,094 (2%) |
| Male genital organs | 277 (0.3%) | 12 (0.2%) | <5 |  |
| Lower female genital tract | 274 (0.3%) | 14 (0.2%) | <5 |  |
| Upper female genital tract | 1,128 (1%) | 41 (1%) | 9 (0.3%) | 1,178 (1%) |
| Skin | 297 (0.3%) | 11 (0.1%) | <5 | 310 (0.3%) |
| Soft tissue | 10,594 (10%) | 834 (11%) | 191 (7%) | 11,619 (10%) |
| Bones and joints of skull and spine | 2,729 (3%) | 228 (3%) | 145 (5%) | 3,102 (3%) |
| Other bones and joints | 35,296 (33%) | 2,060 (27%) | 366 (14%) | 37,722 (32%) |
| Miscellaneous operations | 1,786 (2%) | 144 (2%) | 15 (1%) | 1,945 (2%) |

OPCS-4, Office of Population Censuses and Surveys classification system for interventions and surgical procedures

**Table S5.** One-year outcomes excluding those with an operation date between 1 April 2019 and 31 March 2020. Values are median (IQR[range]).

|  | **No steroids**  **(n=1,775,849)** | **Low dose**  **>28 days**  **(n=58,721)** | **High dose > 28 days**  **(n=6,140)** | **Total**  **(n=1,840,710)** |
| --- | --- | --- | --- | --- |
| Number of admissions in year after surgery | 0 (0, 1 [0, 275]) | 1 (0, 2 [0, 168]) | 1 (0, 2 [0, 164]) | 0 (0, 1 [0, 275]) |
| Number of GP consultations in year after surgery | 8 (3, 14 [0, 365]) | 13 (7, 22 [0, 311]) | 12 (5, 21 [0, 158]) | 8 (3, 14 [0, 365]) |
